# Injections of Algesic Solutions into Muscle Activate the Lateral Reticular Formation: A Nociceptive Relay of the Spinoreticulothalamic Tract

**DOI:** 10.1371/journal.pone.0130939

**Published:** 2015-07-08

**Authors:** W. Michael Panneton, Qi Gan, Michael Ariel

**Affiliations:** Department of Pharmacological and Physiological Science, Saint Louis University, St. Louis, MO, United States of America; Toronto University, CANADA

## Abstract

Although musculoskeletal pain disorders are common clinically, the central processing of muscle pain is little understood. The present study reports on central neurons activated by injections of algesic solutions into the gastrocnemius muscle of the rat, and their subsequent localization by c-Fos immunohistochemistry in the spinal cord and brainstem. An injection (300μl) of an algesic solution (6% hypertonic saline, pH 4.0 acetate buffer, or 0.05% capsaicin) was made into the gastrocnemius muscle and the distribution of immunolabeled neurons compared to that obtained after control injections of phosphate buffered saline [pH 7.0]. Most labeled neurons in the spinal cord were found in laminae IV-V, VI, VII and X, comparing favorably with other studies, with fewer labeled neurons in laminae I and II. This finding is consistent with the diffuse pain perception due to noxious stimuli to muscles mediated by sensory fibers to deep spinal neurons as compared to more restricted pain localization during noxious stimuli to skin mediated by sensory fibers to superficial laminae. Numerous neurons were immunolabeled in the brainstem, predominantly in the lateral reticular formation (LRF). Labeled neurons were found bilaterally in the caudalmost ventrolateral medulla, where neurons responsive to noxious stimulation of cutaneous and visceral structures lie. Immunolabeled neurons in the LRF continued rostrally and dorsally along the intermediate reticular nucleus in the medulla, including the subnucleus reticularis dorsalis caudally and the parvicellular reticular nucleus more rostrally, and through the pons medial and lateral to the motor trigeminal nucleus, including the subcoerulear network. Immunolabeled neurons, many of them catecholaminergic, were found bilaterally in the nucleus tractus solitarii, the gracile nucleus, the A1 area, the CVLM and RVLM, the superior salivatory nucleus, the nucleus locus coeruleus, the A5 area, and the nucleus raphe magnus in the pons. The external lateral and superior lateral subnuclei of the parabrachial nuclear complex were consistently labeled in experimental data, but they also were labeled in many control cases. The internal lateral subnucleus of the parabrachial complex was labeled moderately. Few immunolabeled neurons were found in the medial reticular formation, however, but the rostroventromedial medulla was labeled consistently. These data are discussed in terms of an interoceptive, multisynaptic spinoreticulothalamic path, with its large receptive fields and role in the motivational-affective components of pain perceptions.

## Introduction

Chronic muscle and joint pain afflicts many, especially the aged [1]. Indeed, patients’ complaints of fibromyalgia [2] and arthritis are on the rise as the general population ages. Moreover, the aging population is more prone to take prescribed statin drugs, often resulting in muscle pain [3] that is dull and poorly localized. The smallest fibers innervating muscle (fiber groups III and IV) are thought to relay nociceptive signals [4, 5]. Indeed, 58% of fibers innervating the sternocleidomastoid muscle are unmyelinated, and 60% of these may be sensory fibers [6]. C and Aδ fibers end in muscle as free nerve endings [4, 7, 8]; similar small fibers innervate joints [9], 80% of which are c-fibers.

Intramuscular injections of chemical algesic solutions [10], including capsaicin [5, 11], low pH solutions [12] and hypertonic saline [13, 14] are painful to humans. Kaufman and colleagues [11] and others [5, 15, 16] have shown that capsaicin stimulates mostly c-fibers from muscle by activating TRPV1-channels, while low pH solutions activate acid-sensing ion channels (ASICs) [10, 17, 18] found on muscle afferent fibers [15]. The TRPV1 receptor is also sensitive to H^+^ ions and heat but the mechanisms underlying pain from hypertonic saline solutions must yet be determined [10]. While these noxious stimuli activate the peripheral fibers innervating muscle, activation of neurons within the central nervous system after application of algesic solutions to muscles has received but limited attention [19, 20, 21, 22]. Moreover, the routes whereby noxious stimuli applied to muscles reaches levels of consciousness have not been explored.

For many years, students learned of two major afferent pain systems, the spinothalamic and spinoreticulothalamic pathways. However, studies of the spinothalamic tract greatly predominate in the research literature, with an order of magnitude more publications than the spinoreticulothalamic tract. Nevertheless, it is known that the neospinothalamic tract arises mostly from neurons in laminae I and IV-V of the spinal cord, projects directly to the lateral thalamus, contains fibers generally with relatively small receptive fields, and is involved in the sensory-discriminative components of pain; whereas the paleospinothalmic tract arises from neurons in deeper laminae of the spinal cord, projects mostly to medial intralaminar thalamic nuclei, generally contains fibers with very large receptive fields, and serves the motivational-affective components of pain [23, 24].

The multisynaptic spinoreticulothalamic tract on the other hand arises from spinal neurons in laminae IV-V and VII-VIII, projects generally to neurons in medial nuclei of the medullary and pontine reticular formation usually via collaterals of spinothalamic tract neurons [25] but also to the parabrachial complex and catecholamine cell groups, and serves the motivational-affective components of pain [23, 24]. However, the two brainstem structures proposed as relay nuclei of the spinoreticulothalamic tract, the precerebellar lateral reticular nucleus (LRt) in the caudal ventrolateral medulla and the medial pontobulbar reticular formation [23, 24, 26] have few projections to the medial thalamus [27]. Newer data however suggest an area just lateral to the LRt, designated the caudalmost ventrolateral medulla, contains many neurons responsive to nociceptive signals (see [28, 29], for reviews).

Other studies have elucidated a route for noxious stimuli traveling in the spinoreticulothalamic tract to the medial thalamus. A neurophysiological report [30] showed numerous neurons in the internal lateral subnucleus of the parabrachial complex (PBil) responded to noxious cutaneous input of large body receptive fields with prolonged spike activity, prompting the authors to suggest that perhaps PBil neurons are important for relaying chronic pain. This speculation is supported by neuroanatomical studies [31, 32] that showed numerous projections from the PBil to the intralaminar nuclei in the medial thalamus, especially the paracentral nucleus. However, afferent fibers from central neural structures to the PBil were unknown until Sun and Panneton [28] showed a major projection from the caudalmost ventrolateral medulla, juxtaposed lateral to the LRt. Panneton and colleagues subsequently also showed numerous projections from the medullary lateral reticular formation (LRF) [29] to the PBil. Indeed, neurons in the LRF are activated by capsaicin injections into the temporalis muscle [29] as well as by noxious stimulation of whole body cutaneous receptive fields, muscle and viscera [33].

In the present study, the gastrocnemius muscle (GCM) was injected with either the control or an algesic solution known to cause pain in humans. Activated neurons were labeled in the spinal cord and brainstem by c-Fos immunohistochemistry. Unlike the effect of skin stimulation, we show that few neurons were activated with c-Fos in the superficial dorsal horn of the spinal cord and that most of those activated neurons were in laminae IV-V, VI-VII and X. We tested the hypothesis that the density of activated brainstem neurons would be higher in animals who received algesic injections as compared to those with control injections, as well as higher in LRF as compared to the medial bulbar reticular formation. Our statistical analysis supported that hypothesis, suggesting that the LRF serves as a relay of muscle algesia through the pons and medulla. We speculate that activation of LRF neurons after injection of algesic solutions into the GCM supports a relay of the spinoreticulothalmic pathway in the LRF versus its medial counterpart. Moreover, connections from both the LRF and caudalmost ventrolateral medulla to the PBil [28, 29] could provide an indirect route to the medial thalamus and relay to cerebral cortex for a perception of poorly-localized dull pain emanating from muscles.

## Materials and Methods

Twenty-four Sprague Dawley rats (275–299g) were purchased commercially (Harlan Laboratories, Indianapolis, IN) and studied using a protocol approved by the Saint Louis University Animal Care committee in accordance with the guidelines of the National Institutes of Health Guide for Care and Handling of Laboratory Animals. The number of animals used and their pain and suffering were minimized.

Rats were anesthetized briefly (< 1min) with 4% isoflurane and the right GCM injected with 300 μl of either an algesic substance (6% hypertonic saline [n = 6], pH 4.0 acetate buffer [n = 6], 0.05% capsaicin [0.5 mg ml [n = 6]) or the control phosphate buffered saline [pH 7.0; n = 6] using a 27g hypodermic needle. The GCM was chosen because this large muscle is easy to inject, is utilized in numerous studies, and the central projections of its primary afferent fibers have been described [34]. The rats immediately awoke and remained in their home cages for two hours.

### Tissue Processing

After treatment, rats were anesthetized deeply with an intraperitoneal injection of sodium pentobarbital (40mg/kg, Sleepaway Fort Dodge, IA) and perfused through the heart using a peristaltic pump first with saline mixed with 0.25% procaine hydrochloride and then with the fixative (4% paraformaldehyde and 3% sucrose in 0.1 M phosphate buffer [PB; pH 7.3]). The brains were refrigerated in 20% sucrose in PB for days then blocked in the transverse plane using a precision brain slicer prior to cutting frozen sections (40μm) with a microtome. A 1:3 series of sections was collected in PB.

The sections were incubated overnight with antibodies against c-Fos (rabbit polyclonal IgG for c-Fos p62; 1:20,000; Santa Cruz Biotechnology, Santa Cruz, CA) and then reacted immunohistochemically. Sections were washed 3X with 0.1 M PB for 10 min, and then in 0.1 M PB with 0.3% triton for at least 5 min, then soaked for 1hr in goat anti-rabbit biotinylated secondary IgG (1:500; Vector Labs), washed again, and then incubated in an ABC complex (Vectastain Elite; 1:200; Vector Laboratories, Burlingame, CA) for another hour, washed in 3 rinses of PB, and reacted with diaminobenzidine dihydrochloride (DAB) intensified with nickel ammonium sulfate for 4–10 min. Hydrogen peroxide (0.06%) catalyzed the reaction. A series of sections (one each from a case injected with capsaicin, low pH, and 6% saline) also were subsequently double stained with antibodies directed against tyrosine hydroxylase (1:10,000; Immunostar, Hudson, WI), but the intensification process using nickel ammonium sulfate was eliminated. The sections from all cases then were rinsed, mounted on gelatinized slides and air-dried. They subsequently were counterstained with Neutral Red, dehydrated in alcohols, defatted in xylenes, and coverslipped with Permount.

### Nuclear identification

The reticular formation has diffuse organization making nuclear areas difficult to define. For the present experiments, we define the term lateral reticular formation (LRF) to include the following areas in a rat atlas [35]: the medullary reticular nucleus dorsalis (MdD) in the caudal medulla, the parvicellular reticular nucleus (PCRt) in the rostral medulla, and the PCRtA in the caudal pons [35]. The LRF as we defined is analogous to the lateral tegmental fields (LTF) defined by others [36]. By those definitions, there is slight overlap of the ventral LRF with the lateral extremes of the rostral and caudal ventrolateral medulla [28, 34]. However, similar neurons are found surrounding the trigeminal motor nucleus, including the nucleus subcoeruleus medially (SubCD and SubCV [35]) and the intertrigeminal nucleus [35, 37] laterally; these areas also are included in our LRF. We define the medial reticular formation to include four areas: the nuclei reticularis gigantocellularis (Gi), its dorsal subparts in the medulla (DPGi), reticularis pontis (PnC) and dorsomedial tegmental area (DMTg) in the pons.

### c-Fos quantification

**Fig 1:**
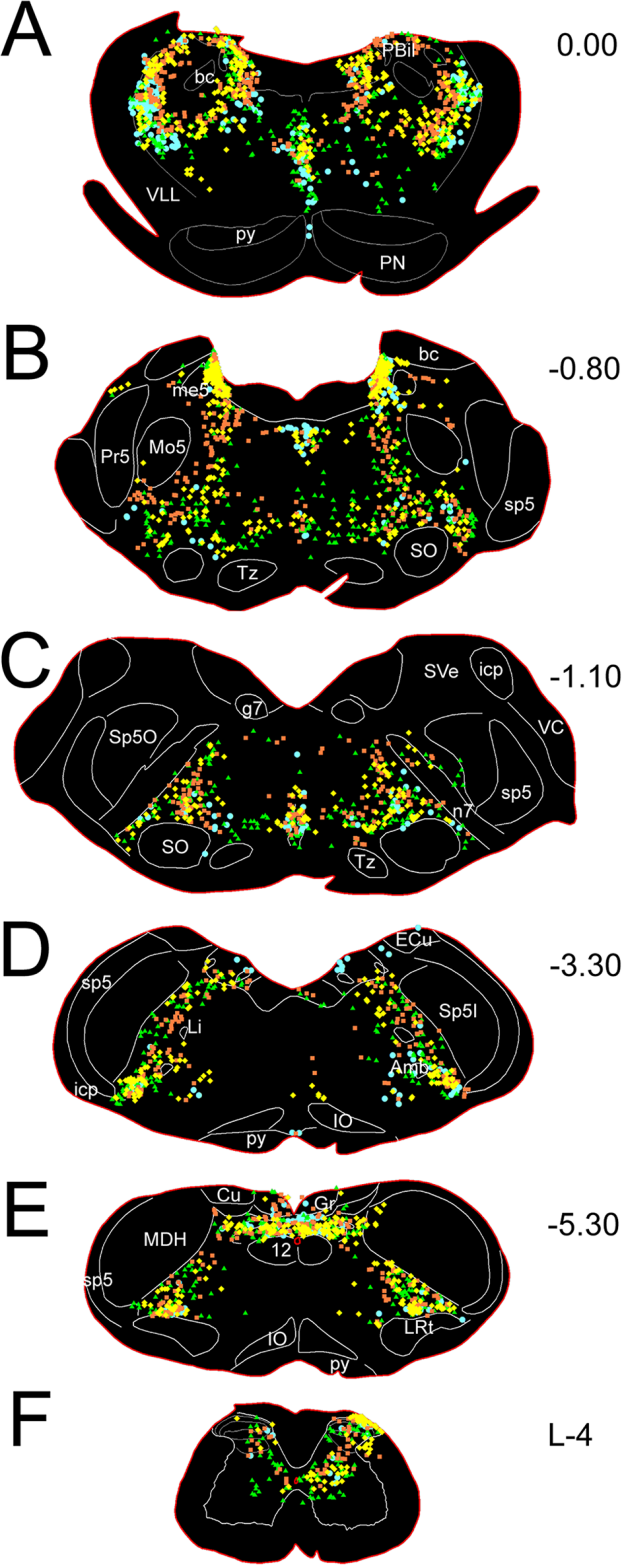
Line drawings illustrating c-Fos activated neurons after a unilateral injection of an algesic solution into the right GCM. Symbols represent a single labeled neuron after injections of buffered normal saline (blue circles), capsaicin (yellow diamonds), low pH acetate buffer (green triangles), and 6% hypertonic saline (red squares). Note the preponderance of immunolabeled neurons in the lateral reticular formation after injections of these algesics into muscle. Also note that immunoreactive neurons in the spinal trigeminal nucleus, parvocellular lateral reticular nucleus and pontine nuclei are not drawn. Numbers to the right of figures represent mm ± 120μm from interaural zero. Abbreviations found on figures:

Sections from all experiments were examined with a Nikon E800 microscope equipped with brightfield and fluorescent optics, photographed digitally (Olympus 41BX microscope with DP-72 digital camera; Research Microscopy Core, Saint Louis University), and processed and saved on a computer. Composite pictures of whole sections were stitched using functions in Microsoft ICE (Microsoft Image Composite Editor; http://research.microsoft.com/en-us/um/redmond/groups/ivm/ice/). The reader may magnify these photomontages to see all of the data from these sections. The location of neurons labeled with c-Fos was reconstructed for Fig 1 using a Neurolucida System (MicroBrightField, Cochester, VT) interfaced with a Nikon E600 microscope. Four experiments were selected with strong labeling contrast: a control case and three with an injection of each algesic into the gastrocnemius muscle. Labeled neurons from those brains were superimposed onto standard brain sections available to our laboratory. Photomicrographs were adjusted in Adobe Photoshop CS2 software using levels, brightness and contrast, and figures were labeled in Adobe Illustrator CS2 software (Adobe Systems, San Jose, CA).

**Table:** 

A1	-	noradrenergic cell group of caudal medulla
A5	-	noradrenergic cell group in ventrolateral pons
Amb	-	nucleus ambiguus
AP	-	area postrema
cmVLM	-	caudalmost ventrolateral medulla
Cu	-	cuneate nucleus
ECu	-	external cuneate nucleus
GiA	-	gigantocellular reticular nucleus, pars alpha
Gr	-	gracile nucleus
IO	-	inferior olivary nucleus
LC	-	nucleus locus coeruleus
Li	-	linear nucleus of medulla
LRF	-	lateral reticular formation of brainstem
LRt	-	lateral reticular nucleus
MDH	-	medullary dorsal horn
Me5	-	nucleus of the mesencephalic tract of the trigeminal nerve
Mo5	-	motor trigeminal nucleus
MRF	-	medial reticular formation
MVe	-	medial vestibular nucleus
PBil	-	parabrachial nucleus, internal lateral subnucleus
Pr5	-	principal trigeminal nucleus
Pn	-	pontine nuclei
RMg	-	raphe magnus nucleus
RVLM	-	pressor area of the rostral medulla
Sol, NTS	-	nucleus tractus solitarii
SO	-	superior olivary nucleus
Sp5I	-	nucleus of the spinal tract of the trigeminal nerve, interpolar part
Sp5O	-	nucleus of the spinal tract of the trigeminal nerve, oral part
SRD	-	subnucleus reticularis dorsalis
SVe	-	spinal vestibular nucleus
Tz	-	trapezoid nucleus
VC	-	ventral cochlear nucleus
VLL	-	ventral nucleus of lateral leminiscus
VMM	-	ventromedial medulla
bc	-	brachium conjunctivum
g7	-	genu of facial nerve
mcp	-	middle cerebellar peduncle
m5	-	motor root of 5n
n7	-	facial nerve
py	-	pyramidal tract
sol	-	tractus solitarii
sp5	-	spinal tract of the trigeminal nerve
7	-	facial motor nucleus
7n	-	facial nerve root
12	-	hypoglossal motor nucleus

### Statistical analysis of LRF labeling

Data at four levels of the brainstem (rostral-pons, caudal-pons, rostral–medulla, caudal-medulla) were analyzed using Neurolucida in six cases (three control and three experimental, a total of 24 sections). The outline of each level was drawn at a low optical magnification, without regard to any c-Fos labeling. Then, outlines of the left and right LRF and MRF were drawn at that same low magnification. Finally, each section was examined at high magnification to mark the position of neurons labeled with c-Fos without regard to nuclear boundaries.

A naïve experimenter quantified the results using the raw counts of labeled c-Fos cells in either the LRF or MRF; the number of labeled cells per 40 μm thick section and the density of labeled cells per mm^2^ were also calculated. Those data were analyzed by a two sample t-test for the statistical difference of their means using a 98% criterion.

## Results

The locations of neurons immunoreactive to c-Fos after unilateral injections of various solutions into the GCM are shown (Fig 1).

### Control Cases

In control cases, after a GCM was injected with normal saline, there were few immunolabeled neurons with c-Fos antibodies in the spinal cord but some were labeled in the brainstem (Fig 1, blue circles). Specifically, cells of the nucleus tractus solitarii, especially its dorsolateral and medial parts near the obex, were consistently labeled in all cases, as were neurons in the A1 area, the parvicellular lateral reticular nucleus, the CVLM and RVLM, the spinal vestibular nucleus, the superior salivatory nucleus, the nucleus locus coeruleus, the A5 area, the nucleus raphe magnus in the pons [35], the pontine grey–including the dorsal tegmental nucleus, the pontine nucleus, and the Kölliker-Fuse nucleus. Neurons in the reticular formation medial to the motor trigeminal nucleus (included in the subcoerulear network), and the superior lateral, external lateral and internal lateral subnuclei of the parabrachial complex were labeled in approximately half of the control cases. There also was labeling in the cochlear nuclei in most of the control cases and immunolabeling in the nucleus ambiguus and the external cuneate nucleus in single cases. Labeled neurons also were seen in the trigeminal complex in all cases but these had random somatotopy, perhaps due to grooming behaviors that rats sometimes perform prior to the terminal anesthesia. Common to these control studies however, was a general dearth of labeled neurons in the reticular formation. Similarly labeled neurons from experimental animals were not included in Fig 1 since they also match control data from previous studies [29, 38], despite different control paradigms.

### Spinal Cord

**Fig 2:**
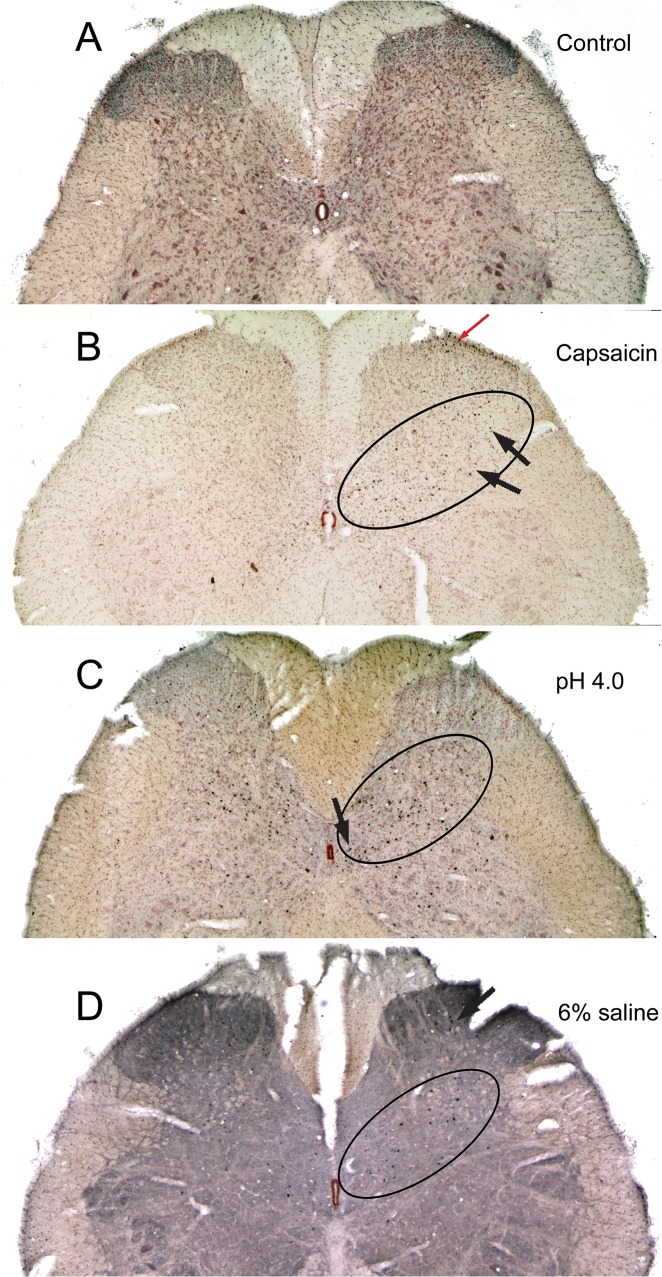
Photomontages of the lumbar spinal cord after a unilateral injection of an algesic solution into the right GCM. Four examples of experiments after unilateral injections of normal saline (A), capsaicin (B), low pH buffer (C), and hypertonic saline (D) into the GCM. Note the abundance of c-Fos immunopositive profiles in intermediate regions of the spinal cord (ovals) after injections of algesic solutions into the GCM. Also note the dearth of neurons within superficial laminae of the dorsal horn after these injections. Arrows illustrate differences in label following injections of different algesic solutions (see text).

c-Fos immunolabeling in the lumbar spinal cord was found generally in deep laminae, IV-VII and X, in all cases after injections of algesic solutions into the GCM (Fig 2, ovals). However, a few neurons in laminae I and outer parts of II also were labeled after capsaicin injections (Fig 2B, red arrow). Numerous neurons also were labeled in lamina III after injections of 6% saline (Fig 2D, arrow), and injections of acidic solutions immunostained more neurons in lamina X (Fig 2C, arrow). It is of interest that the lateral spinal nucleus was considerably more labeled after the capsaicin injections (Fig 2B, black arrows) than injection of the other two algesic solutions (see S1 Fig).

### Brainstem

**Fig 3:**
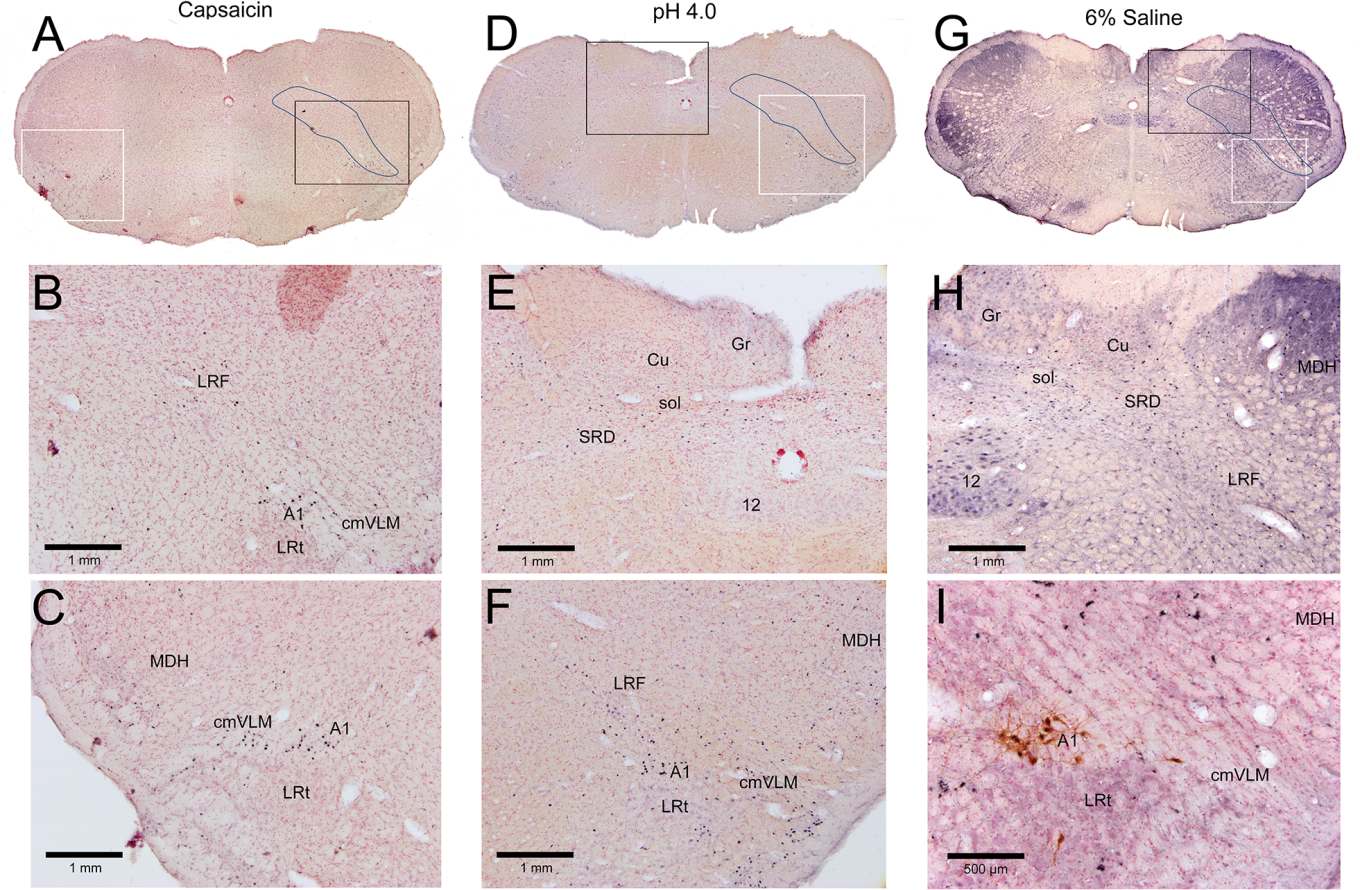
Micrographs of sections through the caudal medulla (between 5.18–5.42 mm caudal to interaural zero) after a unilateral injection of an algesic solution into the right GCM. Each column is headed by a photomontage (A, D, G) of a complete section of a case after injection of capsaicin (A-C), low pH (D-F), or 6% saline (G-I). Many reactive neurons were found in the lateral reticular formation from the caudalmost ventrolateral medulla angling dorsomedially towards the nucleus tractus solitarii (A, D, G; outlined), adjacent to the contours of the intermediate reticular nucleus. Such labeled neurons thus were found in the caudalmost ventrolateral medulla (B, C, F), dorsal medullary reticular formation (labeled LRF; B, C, F, H), and subnucleus reticularis dorsalis (E, H), forming a diffuse band of neurons activated by these different algesic solutions. The larger profiles just dorsal to the lateral reticular nucleus (B, C, F) often were double-labeled with antibodies against tyrosine hydroxylase (F) and thus are labeled the A1 group of catecholamine neurons. Black boxes in A, D, and G represent areas magnified in B, E, and H; white boxes represent areas magnified in C, F, and I.

**Fig 4:**
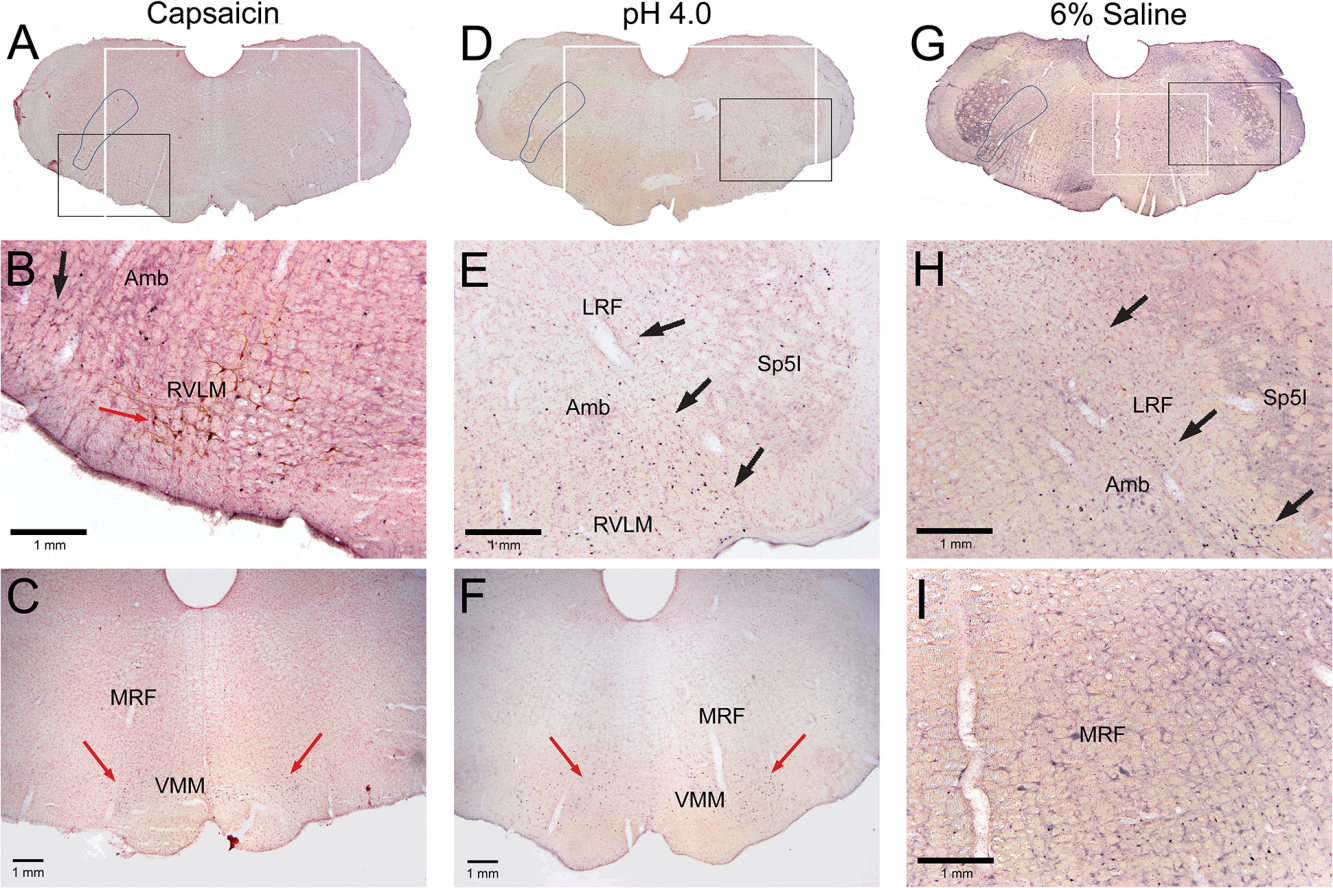
Micrographs of sections through the rostral medulla (between 3.18–3.42 mm caudal to interaural zero) after a unilateral injection of an algesic solution into the right GCM. Each column is headed by a photomontage (A, D, G) of a complete section of a case after injection of capsaicin (A-C), low pH (D-F), or 6% saline (G-I). Similar to the caudal medulla, labeled neurons extended from the ventrolateral medulla through the lateral reticular formation (outlined in A, D, G; arrows in E, H). Other immunoreactive profiles were found in the rostroventrolateral medulla (B, E), but only some were double labeled with tyrosine hydroxylase (B, red arrow). Numerous labeled profiles also were labeled in the ventromedial medulla (C, F; red arrows). However, very few labeled neurons were found in the medial reticular formation in the medulla (C, F, I). Black boxes in A, D, and G represent areas magnified in B, E, and H; white boxes represent areas magnified in C, F, and I.

**Fig 5:**
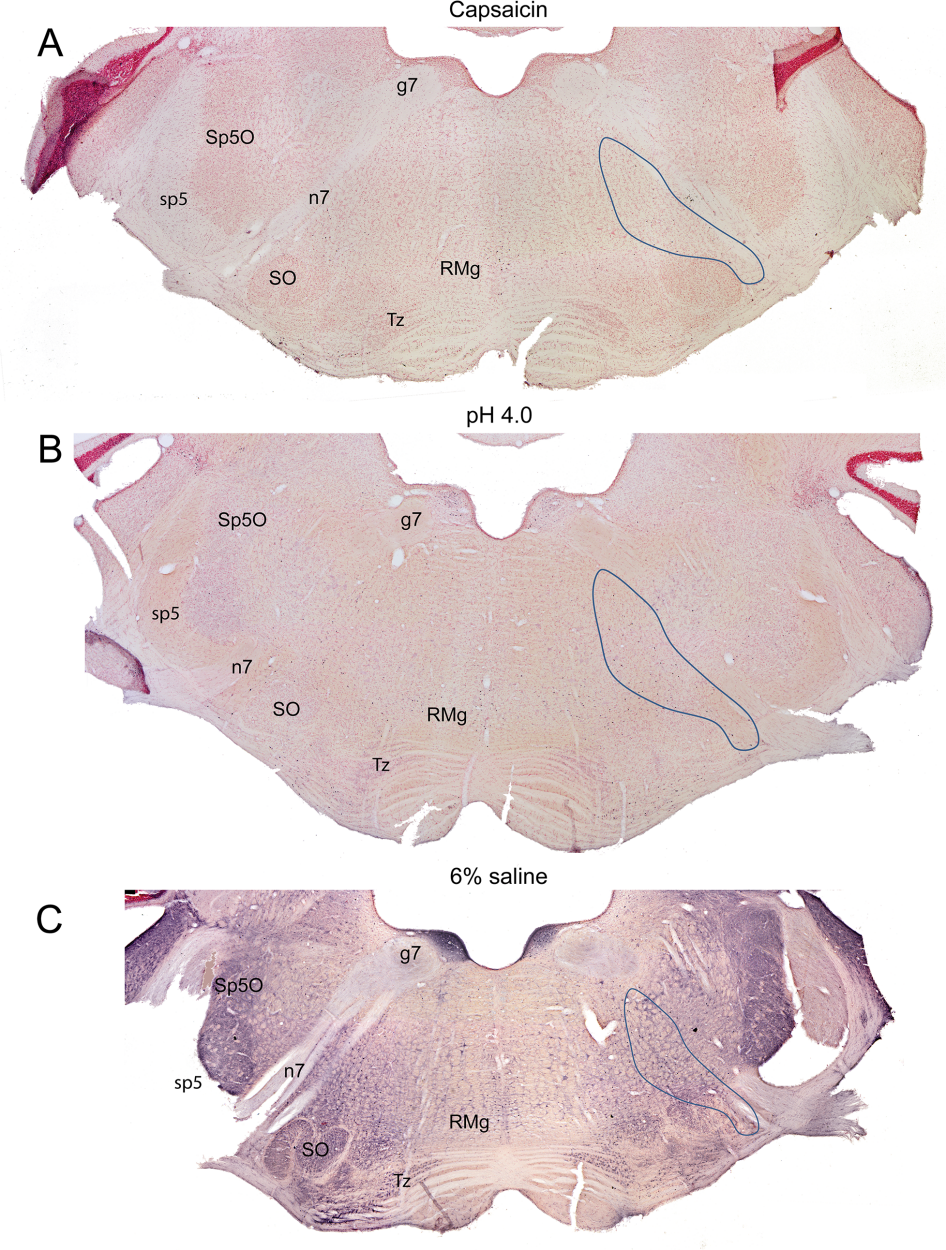
Photomontages of the caudal pons (between 0.92–1.16 mm caudal to interaural zero) after a unilateral injection of capsaicin (A), low pH (B), or 6% saline (C) into the right GCM. The areas outlined on the right of the sections demarcate the LRF as defined by the authors; note the presence of immunoreactive profiles in the LRF bilaterally. Other areas showing c-Fos also were labeled in control cases.

**Fig 6:**
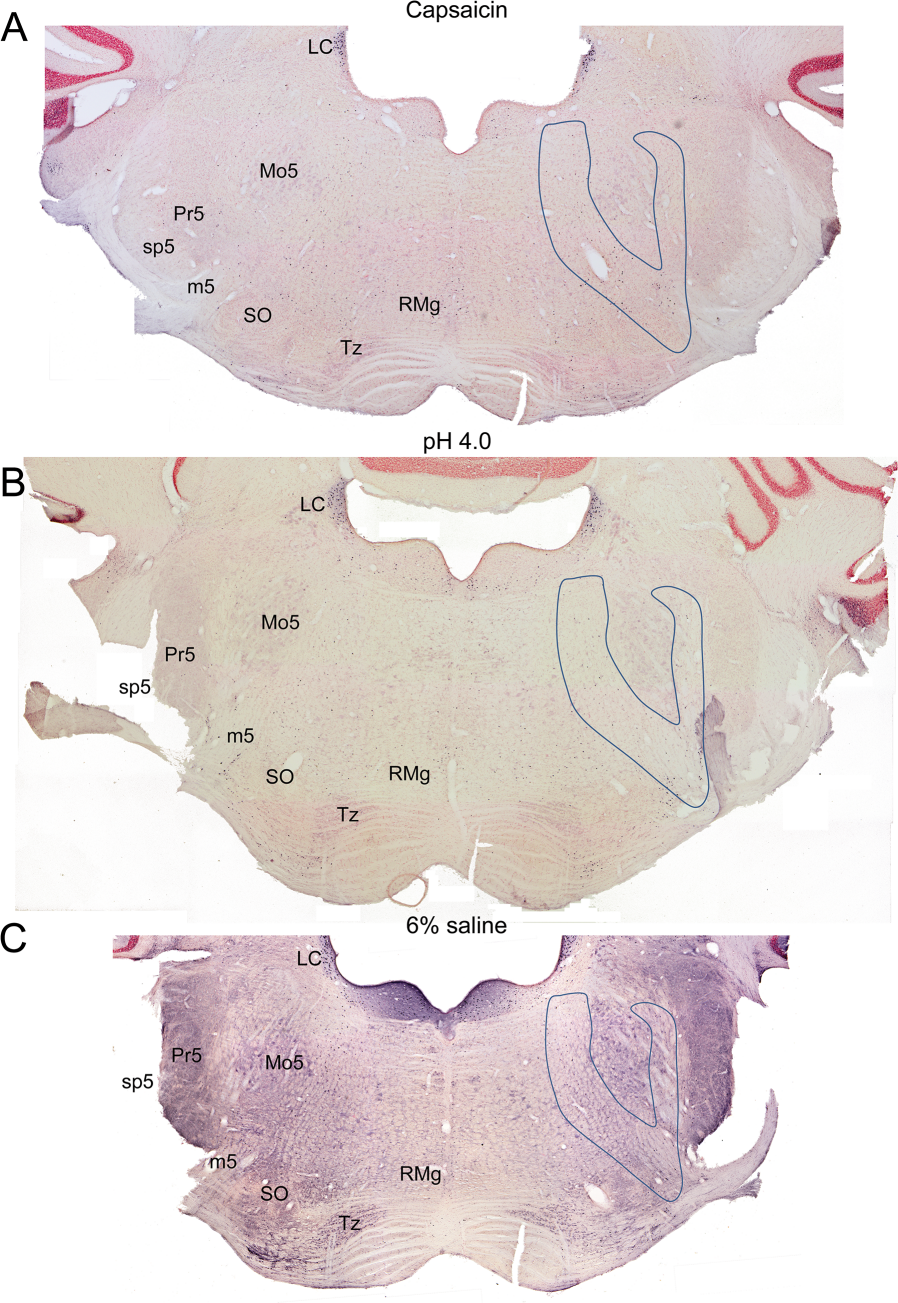
Photomontages at the level of the trigeminal motor nucleus (between 0.18–0.42 mm caudal to interaural zero) after a unilateral injection of capsaicin (A), low pH (B), or 6% saline (C) into the right GCM. The areas outlined on the right of the sections demarcate the LRF as defined by the authors; note the presence of immunoreactive profiles in the LRF bilaterally. Other areas showing c-Fos also were labeled in control cases.

Immunolabeled neurons with antibodies against c-Fos were increased greatly after injections of algesic solutions into the GCM in the brainstem, especially in an area we designate as the lateral reticular formation. This area designated as the LRF is outlined in Figs 3, 4, 5 and 6. In the caudal medulla (Fig 1E, Fig 3), a high percentage of neurons immunolabeled with c-Fos just dorsal to the lateral reticular nucleus also were catecholaminergic (Fig 3I) and these labeled neurons were in the A1 group. Increased immunolabeling with c-Fos was present in the caudalmost ventrolateral medulla (cmVLM; Fig 3B, 3C and 3F), just lateral to the A1 catecholamine cell group (Fig 3I). In contrast, neurons in the magnocellular lateral reticular nucleus (LRt) were labeled rarely. A high density of labeled cells were observed throughout the LRF (Fig 3B, 3F and 3H) along a band called the intermediate reticular nucleus [35]; they then continued into the subnucleus reticularis dorsalis (SRD) and lateral aspects of the solitary complex (Fig 3E and 3H). Neurons in the gracile nucleus (Gr) were labeled bilaterally, as were neurons in the external cuneate nucleus which continues more rostrally.

At more rostral medullary levels (Fig 1D, Fig 4), qualitatively more neurons in the rostral and caudal ventrolateral medulla were labeled with c-Fos following injections of algesic solutions as compared to controls, and a small percentage of them were catecholaminergic (Fig 4B) in our limited sampling of double-labeled neurons. Most impressive was a loose ‘accumulation’ of labeled neurons ventral and lateral to the compact formation of the nucleus ambiguus (Figs 1D; 4B, 4E and 4H; black arrows). More dorsally, neurons were immunolabeled medial to the spinal trigeminal complex (Fig 4E and 4H), although they were sparser in number. Such label was reduced greatly through levels of the facial motor nucleus. All experimental cases were marked with increased numbers of c-Fos immunolabel in the ventromedial medulla through levels of the facial motor nucleus (Fig 4C and 4F; red arrows), including the nucleus raphe pallidus, nucleus raphe magnus, and gigantocellular reticular nucleus, pars alpha. Very few neurons were immunolabeled in the medial reticular formation (MRF; Figs 1D; 4C, 4F and 4I), which consists mostly of the gigantocellular reticular nucleus at this level.

The caudal pons (Fig 1C) showed numerous immunolabeled neurons capping the dorsal and lateral aspects of the superior olivary nucleus; fewer reactive profiles also appeared in control animals. Some of these neurons were also catecholaminergic and part of the A5 group of neurons. The reactive neurons dispersed in the LRF (Fig 5A–5C; outlines) were adjacent to the intermediate reticular nucleus [35]. Neurons in the medial reticular formation, including mostly the caudal pontine reticular nucleus, were seldom labeled.

**Fig 7:**
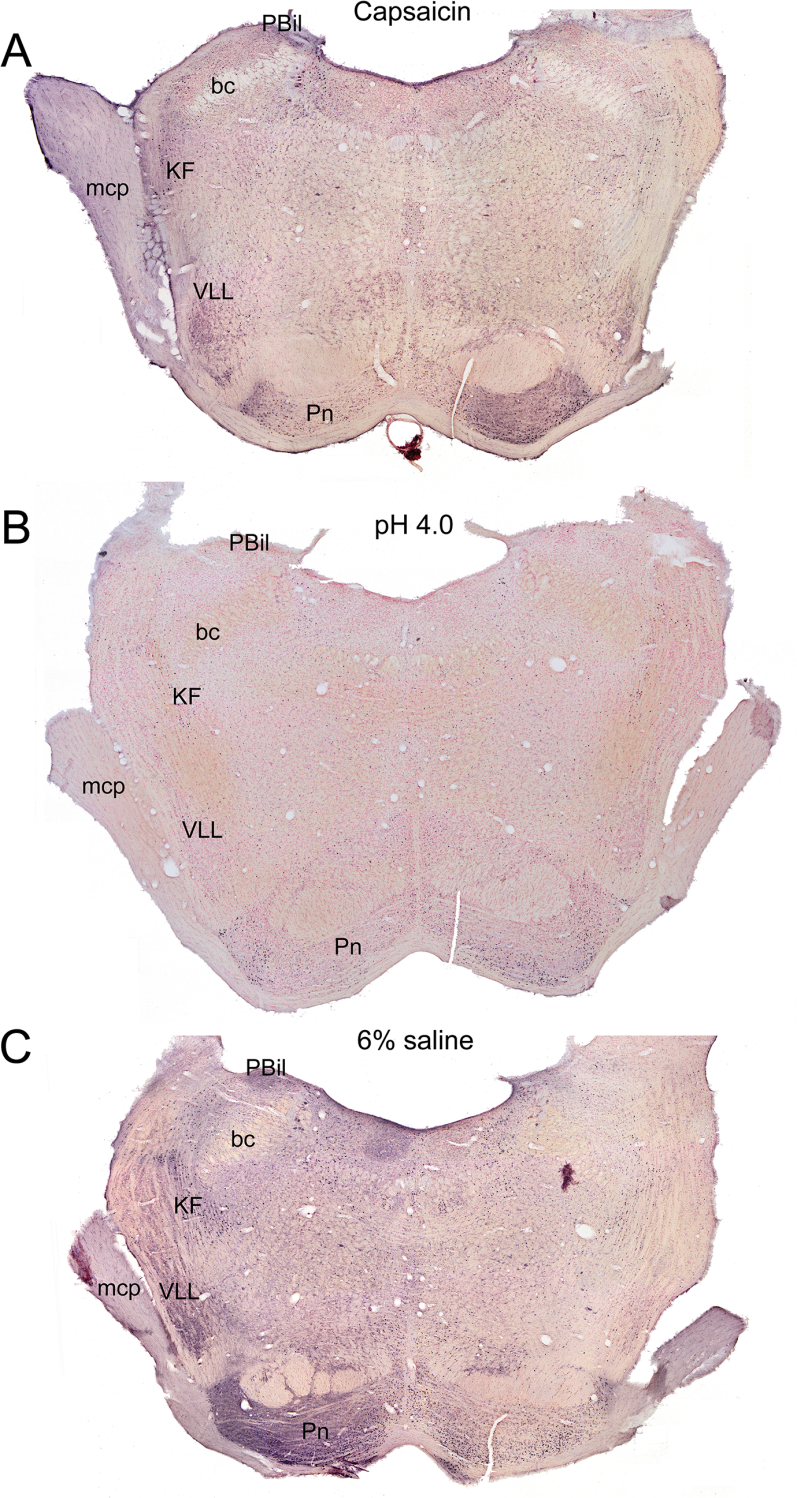
Photomontages at the level of the parabrachial complex (within 0.12 mm of interaural zero) after a unilateral injection of capsaicin (A), low pH (B), or 6% saline (C) into the right GCM. The areas showing immunoreactive neurons above control levels were seen medial to the parabrachial complex angling ventrolaterally towards the ventral nucleus of the lateral leminiscus. Other areas showing c-Fos also were labeled in control cases.

At levels through the trigeminal motor nucleus (Fig 1B), immunolabeling was observed in LRF neurons of experimental animals (Fig 6A–6C; outlines), including parts of the subcoeruleus network [35] medially and the intertrigeminal nucleus laterally. There also were more reactive profiles in the medial reticular formation, mostly in the oral part of the pontine reticular nucleus. Through levels of the parabrachial nuclear complex (Figs 1A, 7), the external lateral and superior lateral subnuclei were consistently labeled in experimental data, but they also were labeled in many control cases. An oblique band of labeled profiles also was seen medial to the parabrachial complex, including the A7 area (not drawn). The internal lateral subnucleus of the parabrachial complex also was labeled, but an abundance of label was seen in only 2 of the 18 experimental cases.

### Quantification of c-Fos labeling

#### Ipsilateral versus contralateral differences

As all injections were made only unilaterally, the density of labeled neurons in the ipsilateral LRF was compared to that of the contralateral side. Combined LRF data from rats injected with algesic substances at four analyzed brain levels (rostral-pons, caudal-pons, rostral–medulla, caudal-medulla) showed no statistical difference between the two sides (p = 0.89). Therefore, density data from both sides were grouped together, resulting in six LRF values measured from the four analyzed brain levels.

#### LRF versus MRF differences

Next, density data in the LRF after algesic injections into the GCM were compared to those of the MRF within the same brains; the difference was statistically significant (p < 0.01). Surprisingly, the LRF-MRF difference was also significant in rats receiving the control saline injection. This finding may indicate that either the LRF has a higher neuronal density than that of MRF or that LRF neurons are more active during basal or sham conditions as compared to those in the MRF.

#### Differences in control versus algesic injections

LRF density changes across the brainstem levels due to each algesic drug was statistically higher than that of saline injections (low pH, p < 0.01; 6% saline, p < 0.01; capsaicin, p < 0.02). In contrast, the same injections did not cause a statistically significant density increase in the MRF. An additional analysis examined whether other brainstem regions besides the LRF also increased their density of labeled cells due to algesic drugs. We found that the total number of labeled cells throughout the brain sections were higher after algesic drugs relative to that of saline injections (p < 0.001). This increase also was observed even after the number of labeled LRF cells were subtracted from that counted in the entire brainstem section (p < 0.002). Therefore, there must be neurons in brainstem regions other than the LRFs that were activated by algesic injections.

A final analysis estimated the relative magnitude of the LRF density increase compared to the density increase of the remaining brainstem areas. Density measurements were expressed as c-Fos labeled cells per mm^2^ of the 40-μm thick brain sections. The density of all labeled cells of the control sections, excluding LRF labeled cells, averaged 1.4 labeled cells per mm^2^. That density value increased by 5.9 labeled cells per mm^2^ due to the algesic injections. In comparison, the density of LRF labeled cells of the control sections had a mean of 9 labeled cells per mm^2^, which increased by 29.9 labeled cells per mm^2^ due to the same algesic injections. This analysis indicates that LRF density increases account for much of the brainstem’s activation even though it spans only 16.2% of the brainstem’s area.

Although increases in labelling density were not observed in MRF, visual inspection of the sections showed large increases in the locus coeruleus (LC), for example, due to injection of algesics drugs. Other nuclei may also have been activated by algesics, but quantification of these brainstem areas were not made because they are small or have diffuse nuclear boundaries.

## Discussion

Pain emanating from deep tissues or viscera was suggested long ago to be different than pain emanating from superficial cutaneous origin [22, 39, 40]. Exteroceptive sensation of nociceptive stimuli applied to skin is generally well-localized and its study emphasizes the spinothalamic pathway as the route for its ascending transmission. In contrast, interoceptive sensations, which strongly influence mood and emotional state while also monitoring homeostasis [41], often are poorly localized and, in some cases, result in a whole body perception of pain, including viscera and muscle. However, studies have shown that the spinothalamic pathway is less important for the central transmission of noxious visceral stimuli than a pathway via the dorsal columns [42, 43] since pelvic cancer pain in humans is successfully alleviated or abolished in patients with a limited dorsal column myelotomy [44, 45, 46]. Thus, deep pain after viscera stimulation may use different ascending paths than those mediating cutaneous pain.

**Fig 8:**
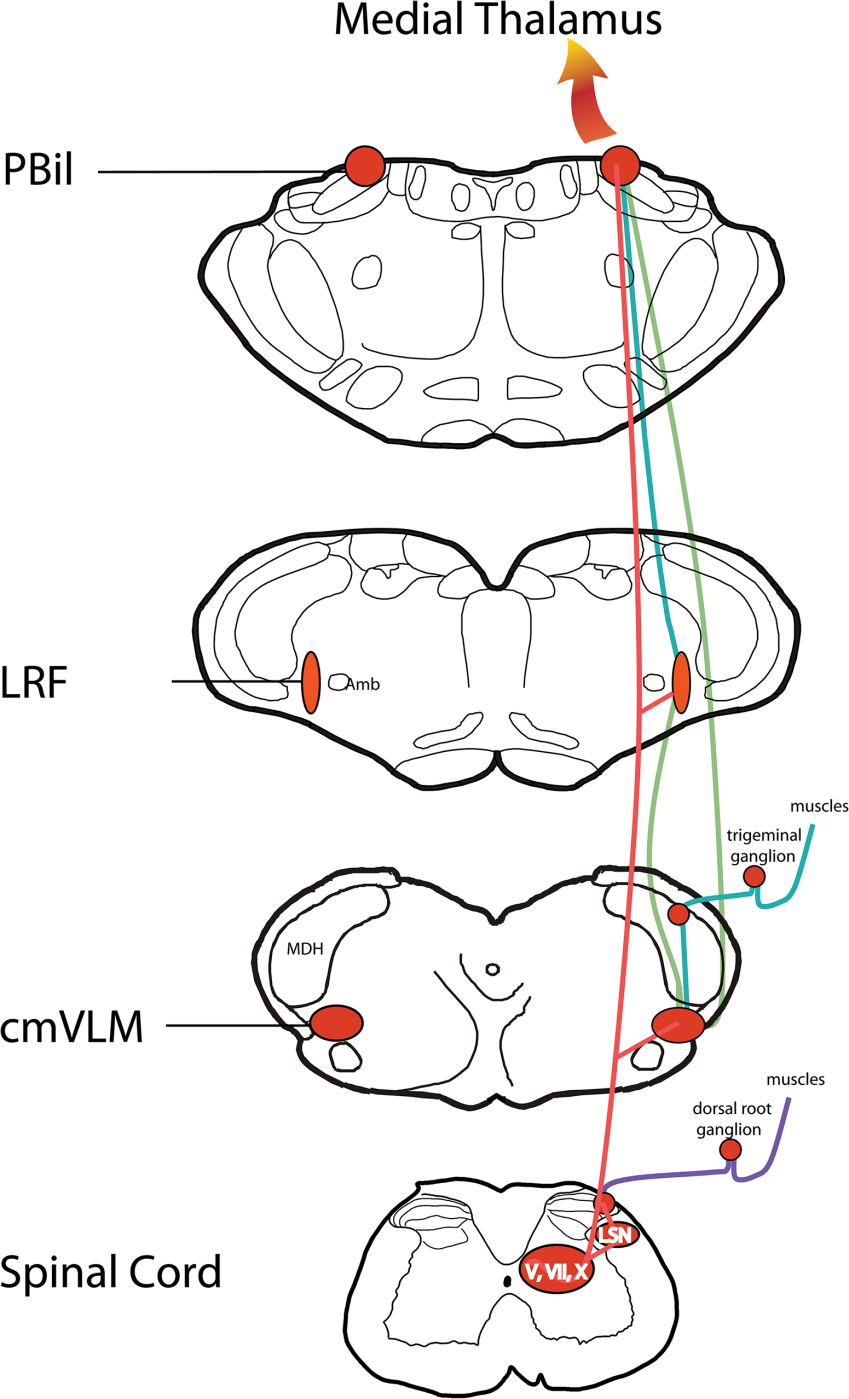
Summary diagram illustrating the multisynaptic spinoreticulothalamic tract. Data that provide components of this tract were garnered from several independent neuroanatomical studies including primary afferent projections of the GCM to lumbar spinal cord (purple lines; [34]) projections from the cmVLM (green lines; [28]), projections from the LRF (blue lines; [29]), and the data presented herein (red lines). We propose this multisynaptic pathway utilizes mostly very small fibers and is important for diffuse deep pain, including that from both muscles and viscera. We also suggest all central neurons part of this system will have very large receptive fields and respond to various multimodal stimuli. Although the multisynaptic path is drawn on the right side only, the projections from all sources are bilateral.

A pattern of central neuronal activation after injections of algesic solutions into the deep GCM was observed that differs from the pattern elicited after injections of algesic chemicals into the skin. Most spinal neurons with c-Fos activated after muscle injections of algesic solutions were found in deep laminae of the spinal cord (e.g., laminae IV-V, VI, VII and X) versus the more superficial location (laminae I- II) found after noxious cutaneous stimulation. In the brainstem, the amount of neuronal activation after muscle injection was substantial in the LRF, an area often ignored in contemporary studies of cutaneous pain. While the functional role of these neurons only can be speculated with the neuroanatomical assays used herein, preliminary electrophysiological studies [33] suggest that LRF neurons respond to algesic stimulation of muscles, to stimulation of the nasal mucosa with noxious vapors, and to stimulation of high threshold skin mechanoreceptors. Also, receptive fields of LRF neurons are large, sometimes responsive throughout the body. Thus, interoceptive sensations induced by noxious muscle stimulation may not be carried by the spinothalamic tract, but by the spinoreticulothalamic tract (Fig 8).

### Technical Limitations

The limitations of the c-Fos technique are widely known [47, 48, 49, 50] and have been discussed specifically with regard to our protocols [29, 38, 51]. An activation of a neuron can be initiated from various stimuli yet not all neurons are immunolabeled by the c-Fos technique. Nonetheless, we feel that using this technique is still worthwhile because its study direct more confirmative investigations. Indeed, the c-Fos technique utilized herein has led to electrophysiological studies that record LRF responses to injections of algesic substances into both the temporalis and GCMs from either side [33].

Chemical nociceptive stimuli were used in this study rather than the more common mechanical probing because they are more analogous to the effects of muscle fatigue induced by exercise. Also, these solutions are applied more easily to awake animals. These substances, albeit non-physiological, are known to activate primary afferent fibers innervating muscle [10, 11, 15, 17, 18, 52, 53]. LRF neurons were activated even though an injection of pH 4.0 solution may be neutralized quickly by buffers in the muscle [54] and the effects of hypertonic saline and capsaicin may only produce acute pain [55]. Perhaps more prominent activation of central neurons would have occurred if substances injected into muscle were combinations of specific agonists of ASIC and TRPV1 receptors found peripherally [56].

### Spinal Cord

The present study showed immunolabeled neurons mainly in laminae IV-V, VI, VII and X of the spinal cord. While the present c-Fos data mimics that presented by two studies focusing on the spinal cord after stimulation of the GCM [19, 21], our data differs markedly from that of other c-Fos studies after algesic injection of muscle. Two studies [57, 58] showed little c-Fos immunoreactivity, while another [59] showed thousands of immunoreactive profiles bilaterally in all spinal laminae. These discrepancies may be due to differing levels of labeling sensitivity that are intrinsic to individual laboratories.

Several studies have shown primary afferent fibers innervating muscle project broadly into lamina I and one showed a dense projection into inner parts of lamina II (see [34] for review); thus neurons in these laminae could have been activated directly by these primary afferent fibers. However the present study showed relatively few c-Fos immunoreactive neurons in laminae I-II after injections of low pH or hypertonic saline into the GCM, but more were found in lamina I and outer parts of lamina II (IIo) after capsaicin injections. Capsaicin is a powerful stimulant, and it is possible that residue on the injection needle may have contaminated skin in route to the deeper muscle. Indeed, there was a small locus of neurons in laminae I-II even in cases with injections of isotonic saline of neutral pH, again suggesting that penetration of the skin by the needle may have induced such labeling. Nevertheless, while some of these afferent fibers may have been excited directly, which in turn could activate lamina I projection neurons, our data suggest that few of those projecting to inner parts of lamina II [34] were activated by the algesic chemicals since few neurons here showed c-Fos immunolabeling. Several neurons in the lateral spinal nucleus also were immunoreactive after these injections, especially after capsaicin injections. Supplementary data (S1 Fig) suggests a projection from neurons in outer portions of lamina II project to neurons of the lateral spinal nucleus, and may have activated them, similar to projections observed in the medullary dorsal horn to the caudalmost ventrolateral medulla [29].

After noxious visceral stimulation, most c-Fos immunoreactive spinal neurons were found in deep laminae [60], similar to the present data after algesic injection of muscle. Primary afferent fibers innervating viscera also have few projections into lamina II [61, 62]. It is of interest that pain from muscle and viscera are both poorly localized, both have minimal projections into outer parts of lamina II, but a strong representation in deep laminae of the spinal cord. Theories on pain processing [63, 64, 65], usually show neurons in lamina II, especially its outer sublamina, as important in mediating nociception, especially that to cutaneous stimuli. Our laboratory has observed several times over many years that primary afferent fibers innervating numerous receptive fields are only diffusely somatotopically organized in laminae I and V but tightly organized in lamina II [34, 66, 67, 68, 69]. We speculate that the tight somatotopy found in lamina II is important for spatial discrimination of noxious stimuli, especially in lamina I nociceptive-specific neurons with small receptive fields. Nociceptive signals from both muscle and viscera, with their limited primary afferent projections into outer parts of lamina II but wide distribution in laminae I and V, relinquishes the modulatory action of lamina II neurons on lamina I projection neurons, with the result being very large receptive fields that might mediate a diffuse aching pain.

The distribution of activated neurons in deep laminae of the spinal cord after algesic injections into muscles mimic that of nociceptive neurons, many of which project to the thalamus [24, 25, 43, 70]. The distribution of spinal neurons labeled in the present study also mimics that of presumed interneurons labeled after injections of pseudorabies virus into the medial gastrocnemius muscle [71], promoting a disynaptic link for muscle reflexes. Supplementary data (S2 Fig) suggest that neurons in this area also send very small axons with varicosities into the LRF as well as the PBil; similar projections are noted from the lateral spinal nucleus. Such evidence reinforces our proposal that these projections are part of a spinoreticulothalamic tract mediating painful sensations.

### Brainstem

The rostroventromedial medulla and raphe nuclei are well-documented as contributing to descending modulation of pain, and neurons in both areas were activated after algesic injection of muscle. Neurons labeled with c-Fos were found in the nucleus gracilis, especially ipsilaterally, after muscle injections and may be similar to those labeled after injections of retrograde tracers into the central lateral thalamic nucleus and responsive to visceral pain [43]. Many neurons in the nucleus tractus solitarii (Sol) also were immunolabeled. It is also of interest that neurons in the lateral aspect of the Sol are morphologically similar to those in the subnucleus reticularis dorsalis, which has been implicated in modulating noxious stimuli. This Sol region receives projections from the cmVLM, an area also implicated in ascending paths of noxious stimuli [28]. Similar Sol neurons in human patients receiving stimulation of the vagus nerve may modulate their pain perception [72, 73].

### Catecholaminergic Neurons

Data above suggest that brainstem catecholamine neurons are commonly activated with c-Fos after muscle injections of algesic solutions. Some neurons in nucleus tractus solitarii were A2 catecholamine neurons, whose function must still be determined [74]. The A1, A5, and A6 (locus coeruleus) groups were especially activated, but there also were some double-labeling of catecholamine neurons in the RVLM (C1), subcoeruleus groups and A7. The few cases presented on activated tyrosine hydroxylase positive neurons after noxious muscle stimulation conforms to that seen after visceral stimulation [75, 76].

### Reticular Formation

While numerous reports show increased c-Fos activity in selected brainstem areas (VMM, raphe nuclei, NTS, catecholamine neurons) after different noxious stimuli to different targets, labeling in the brainstem reticular formation is seldom reported. Neurons in the cmVLM, well-known to be responsive to noxious stimulation of both skin and viscera (see [28, 29] for review), were immunolabeled in the present study suggesting they also respond to noxious stimulation of muscle. The c-Fos-immunolabeled cmVLM neurons overlap with those reported by others who used varied noxious stimuli [77, 78, 79, 80], but these studies all utilized anesthetized animals, often involving surgery. The densest aggregation of c-Fos immunolabeled profiles after GCM injections was in the ventral aspect of the LRF of the medulla caudal to the facial nucleus. That area may correspond with the label generated from anterograde studies after tracer injections in the spinal cord [27] as well as c-fos immunolabeling after muscle stimulation and visceral stimulation [77, 78] but none of those reports indicate the exact location of their photomicrographs within the reticular formation. Our data does overlap considerably with activated neurons in the LRF after capsaicin injection into the temporalis muscle [29]. Nevertheless, the spike responses of numerous neurons in the medullary LRF respond to noxious stimuli, including that applied to muscles, has recently been reported [33]. These neurons generally had very large receptive fields and responded to activation of both cutaneous and muscle nociceptors.

Spinoreticular neurons have been identified electrophysiologically; most have wide receptive fields which respond to high threshold (noxious) stimulation [81, 82, 83]. Moreover, neurons in the medial reticular formation respond to noxious stimuli [81, 82], and the ascending neurons of origin are located in spinal laminae that also are immunolabeled [84]. However, most neurons found in the medial reticular formation, including the nuclei reticularis gigantocellularis and reticularis pontis, project down into laminae VII and VIII of the spinal cord and appear to be better suited to modulate motor neurons in the ventral horn [85, 86]. Nevertheless, most of those activated by injections of algesic solutions into the GCM were found in the LRF. The properties of LRF neurons are similar to those described previously for more medial neurons, e.g., neurons responding to wide-body bilateral receptive fields after both cutaneous and muscle stimulation [33, 87]. Many of the LRF immunolabeled neurons were adjacent to the intermediate reticular nucleus [35] and continued rostrally to surround the trigeminal motor nucleus. While the area designated here as the lateral reticular formation takes on several other designations in a popular atlas [35], the LRF appears more as a continuum to us, perhaps with common functionality.

These labeled LRF neurons also could be part of an ascending reticular activating system, which arouses an organism’s consciousness by increasing awareness of its environment and self. The ascending reticular activating system arises from neurons in the reticular formation and projects through intralaminar nuclei of the thalamus for relay to the cerebral cortex [88]. However most ascending reticular projections arise from brainstem neurons located rostral to the trigeminal nerve entry zone [88], and utilize neurons of the pedunculopontine nucleus, where few neurons were immunolabeled in the present study. In conclusion, the reticular neurons labeled in the present study may be more important in relaying ascending nociceptive messages, perhaps regarding motivational-affective aspects of those signals.

### Parabrachial Complex

The present c-Fos studies showed most parabrachial neurons activated by noxious stimulation were in the dorsolateral and superior lateral subnuclei, similar to other studies where noxious stimuli were applied [89, 90, 91]. Although the lack of robust labeling in the PBil was surprising, the PBil does receive direct brainstem inputs from both the LRF [29] and the caudalmost ventrolateral medulla [28], as well as spinal input from deep laminae in the spinal cord [70, 92, 93, 94, 95, 96],supplementary data]. Moreover, the PBil has numerous connections to intralaminar nuclei of the medial thalamus [31, 32] and has been suggested as a relay of chronic pain [30].

### The Spinoreticulothalamic Tract

It has been proposed that pain from deep organs are relayed through a different pathway than that from the skin [22, 40]. Moreover, spinal projections to medial and lateral thalamus have different places of origin as well as different characteristics [97, 98]. Indeed, physiological and clinical studies suggest that perception is altered whether the medial or lateral thalamus is activated [98]. While numerous pain pathways have been described, they generally are divided into two realms; one group is for fast pain and supplies sensory-discriminative aspects of pain while the other path is for slow pain and interacts with motivational-affective brain circuits [99]. While most agree that the spinothalamic tract to the lateral thalamus imparts sensory-discriminative modalities, a path(s) to motivational-affective circuits is less clear. Many pain researchers surmise that the motivational-affective components are relayed either to the amygdala via collaterals of spinothalamic tract fibers to the parabrachial complex, to the hypothalamus via spinohypothalmaic or spinoparabrachiothalamic tracts, or the spinoreticulothalamic tract. The spinoreticulothalamic tract has long been proposed to be a phylogenetically old pathway with multiple synapses through its ascent. Our descriptions of such a path reflect such multiple synapses, while our evidence suggests this nociceptive relay is mediated in part through neurons in the lateral reticular formation rather than its medial counterparts as suggested previously [23, 25, 26]. We thus propose that the spinoreticulothalamic tract relays polymodal information with large receptive fields in the brainstem via neurons in the cmVLM, LRF, and PBil (Fig 8). This interoceptive pathway could also interact with circuits that monitor homeostasis and motivational-affective responses [41]. Future investigations of this pathway using more refined imaging methods on awake humans might reveal new information about the role of this spinoreticulothalamic tract [100].

## Supporting Information

S1 Fig Photomicrographs illustrating projections from neurons in laminae I-II of spinal dorsal horn to the lateral spinal nucleus (LSN) after a BDA injection. The small injection (A) sends numerous small diameter fibers into LSN (B, C). The areas boxed in A are seen at higher power in B and C. Note the numerous boutons along the small fibers juxtaposed to LSN neurons.
(TIF)

S2 Fig Photomicrographs illustrating spinal projections to the lateral reticular formation (LRF) and the internal lateral subnucleus of the parabrachial complex (PBil). Injections of FluoroGold into the lateral medulla (A) retrogradely-labeled neurons (B, C; arrows) in the LSN and laminae IV-V, VII, and X of the spinal cord. Spinal injections of BDA centered in laminae VI-VII (D; box) induced numerous very small fibers with varicosities in the LRF (E; arrows) and PBil (F; arrows). Moreover, injections centered in the LSN (G; box) also showed similar small fibers in the LRF and PBil (H, I respectively; arrows).
(TIF)
