# Supplementary figures and images for: Injections of Algesic Solutions into Muscle Activate the Lateral Reticular Formation: A Nociceptive Relay of the Spinoreticulothalamic Tract
Source: PLoS One. 2015 Jul 8;10(7):e0130939. doi: 10.1371/journal.pone.0130939 (PMC4496070; doi:10.1371/journal.pone.0130939)

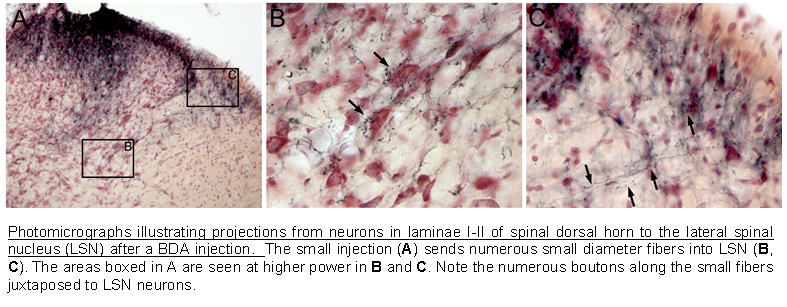

Supplement: S1 Fig — The small injection (A) sends numerous small diameter fibers into LSN (B, C). The areas boxed in A are seen at higher power in B and C. Note the numerous boutons along the small fibers juxtaposed to LSN neurons. (TIF) [file pone.0130939.s001.tif]

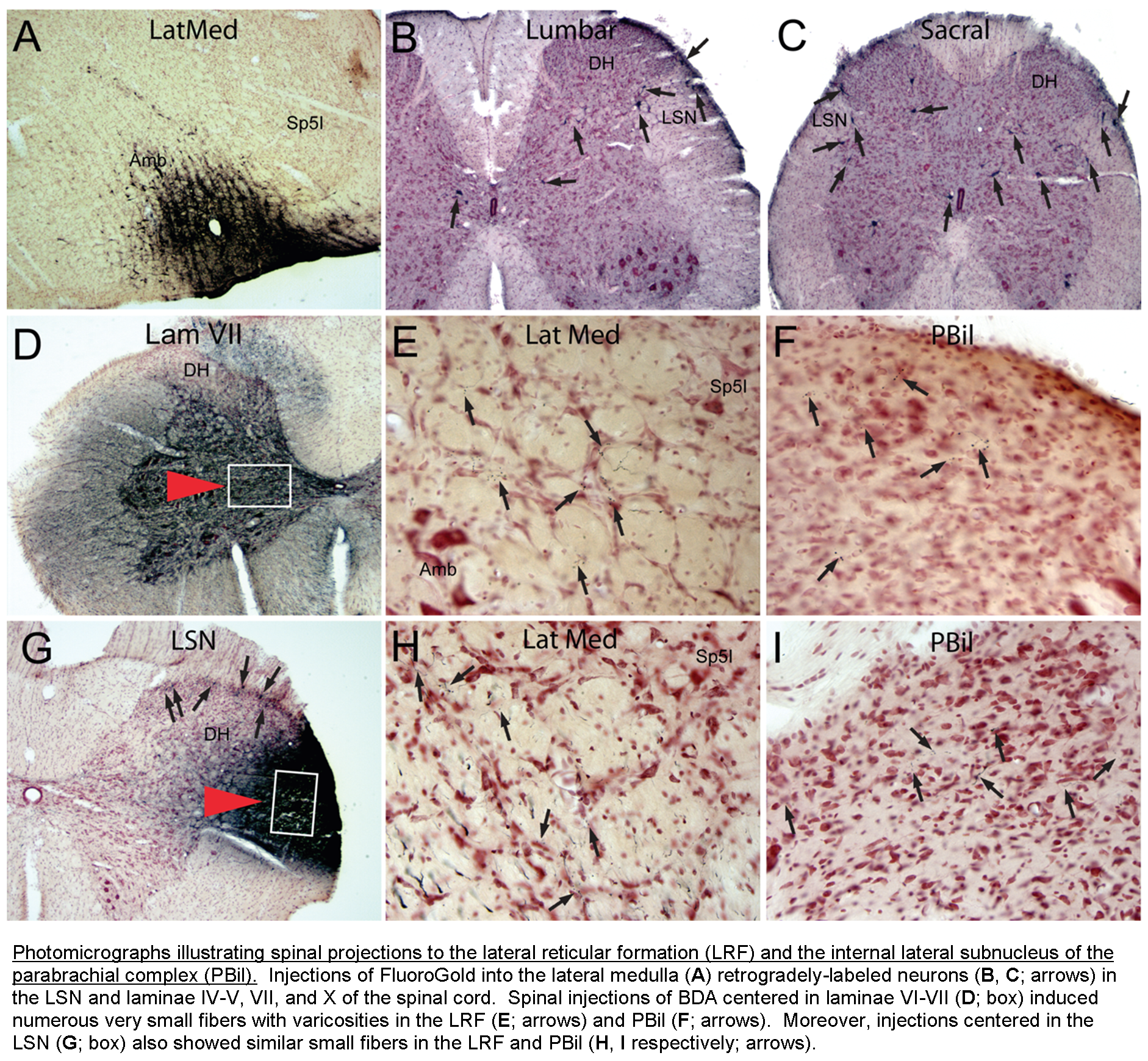

Supplement: S2 Fig — Injections of FluoroGold into the lateral medulla (A) retrogradely-labeled neurons (B, C; arrows) in the LSN and laminae IV-V, VII, and X of the spinal cord. Spinal injections of BDA centered in laminae VI-VII (D; box) induced numerous very small fibers with varicosities in the LRF (E; arrows) and PBil (F; arrows). Moreover, injections centered in the LSN (G; box) also showed similar small fibers in the LRF and PBil (H, I respectively; arrows). (TIF) [file pone.0130939.s002.tif]
